# The difficulties experienced during the preparation and administration of oral drugs by parents at home: a cross-sectional study from Palestine

**DOI:** 10.1186/s12887-020-02105-w

**Published:** 2020-05-07

**Authors:** Ra’fat Ali, Abdullah Shadeed, Hasan Fitian, Sa’ed H. Zyoud

**Affiliations:** 1 Department of Medicine, College of Medicine and Health Sciences, An-Najah National University, Nablus, 44839 Palestine; 2 Poison Control and Drug Information Center (PCDIC), College of Medicine and Health Sciences, An-Najah National University, Nablus, 44839 Palestine; 3 Department of Clinical and Community Pharmacy, College of Medicine and Health Sciences, An-Najah National University, Nablus, 44839 Palestine; 4 Clinical Research Centre, An-Najah National University Hospital, Nablus, 44839 Palestine

**Keywords:** parents, oral medications, administration, children

## Abstract

**Background:**

Failure to properly administer drugs to children at home may cause adverse events, which makes it a challenging job for parents or caregivers. The main goal of this study was to investigate the problems and difficulties that parents or caregivers faced when administering oral drugs to their children at home.

**Methods:**

A cross-sectional study was conducted using a questionnaire consisting of ‘yes/no’ and multiple-response questions to assess parents' experiences and problems with administering medication to their children at home. Data was collected from parents who visited primary health care centres in Nablus. Descriptive analysis was conducted to describe the characteristics of the sample.

**Results:**

We interviewed 420 parents. 91.9% of the parents used drugs without prescription from a doctor, and the most commonly used non-prescription medicines were antipyretics (*n*=386, 100%), influenza drugs (*n*=142, 36.8%), cough drugs (*n*=109, 28.2%) and antibiotics (*n*= 102, 26.4%). The study showed that 21.7% of parents used teaspoon and 7.1% used tablespoon in administering liquid medications to their children. When the children refused taking liquid medications, almost two-thirds of the parents (65.7%) insisted their children take them, 21.5% mixed it with juice, 5.2% mixed it with food and 4.7% mixed it with milk. 12.4% of the parents reported that they gave drugs in doses higher than prescribed by the doctor to treat their children more quickly. Also, our study revealed that 80.5% of the parents gave medications at incorrect intervals.

**Conclusions:**

This study has shown that there is a proportion of caregivers or parents who administer oral drugs to their children incorrectly, which may involve giving them at the wrong intervals or doses, using incorrect instruments, or mixing them with food, juice or milk. The development of educational programs that will provide parents with education about medication administration is therefore recommended.

## Background

The administration of drugs at home may result in errors for the children, made by parents or caregivers, and may occur during administration, or by giving the drugs in the incorrect intervals or doses [1, 2]. The proper administration of drugs requires the calculation of correct doses based on the weight of the child, using the right instruments, slow administration, and respect for the powder weight of drugs [3–5]. Failure to implement these conditions could cause adverse events [6]. Non-prescription or over-the-counter (OTC) drugs have also become excessively widespread in recent years, and have become major issues in children’s health [7–10]. Lack of parental education and poor counselling or pharmacists' inadequate knowledge and confidence make incorrect administration and drug errors more common in developing countries, because outpatient clinics are very crowded and parents or caregivers are not well educated about health [11, 12].

Most currently available solid dosage forms cannot be used as produced in children under the age of 6–7 years, and the need to change dosage forms and dosing presents additional challenges for the pharmacist in the dispensing [13]. To our knowledge, several studies have been conducted among parents [14–18], considering aspects other than the difficulties that the parents experienced in administrating oral medications to their children. Parental difficulties in administering oral medications to their children were not assessed by any of these studies, however, or any other study conducted in Palestine. This study is thus the first conducted in Palestine to evaluate that issue. Medication efficacy may be altered by the use of inappropriate techniques used by the parents to overcome their children’s’ difficulties in swallowing oral medications. Acetaminophen is well established for safe use in children, and the risk of toxic reactions in children is less than in adults, and when it does happen it is mostly due to intentional overdoses [19, 20]. On the other hand, the under-dosing of acetaminophen in pediatric patients with fever may prolong their condition [18]. Oral liquid medications are commonly given by teaspoon, the capacity of which can range from 1.5 ml to 9 ml, leading to dosing errors with the subsequent complications. Little information about using self-therapy antibiotics in children in developing countries is available, despite the high rate of antibiotic resistance.

The health profile for Palestinian children keeps the issues facing the world in the 21st century in microcosm including migration, poverty, conflict, environmental degradation, and inadequate access to health care [21]. Therefore, this study was undertaken mainly to investigate the difficulties experienced by a sample of the Palestinian population in administering oral medications to their children at home. It is hoped that this research will contribute to a deeper understanding of this problem for many reasons. Some medication administration errors are life-threatening, as noted above. The use of the incorrect tools to administer medication leads to incorrect doses which have bad consequences on children’s health. This study will provide a good foundation for further studies in this field. Healthcare providers are encouraged to educate patients about the correct techniques to overcome medication swallowing difficulties. Baseline data will be available from this study for educational purposes.

## Methods

### Study design and setting

This study was designed as a cross-sectional study. The study was conducted in the primary care centres in Nablus. Nablus city was chosen as it is one of the largest cities in Palestine. The data was collected over a period of 3 months from June 2018 to 31 to August 2018.

### Participants

The subjects of our study were selected from people who visited the children care clinic in the primary care centers in Nablus where parents routinely went to vaccinate their children and thus where we concentrated on the dates when the appropriate sample could be recruited. The samples were selected by convenience with inclusion criteria of parents aged more than 19, who had a child or more aged between 6 months and 10 years [22–25], and who agreed to participate in the study. Subjects with vision problems, cognitive/physical disabilities, and caregivers other than parents were excluded. Convenience sampling was used. A total of 434 parents were approached; however, we got a consent from 420 parents with a valid response of 96.8%.

### Intervention (questionnaire)

The data was collected using a data collection questionnaire based on the relevant literature [11, 26–28]. The data collection form included four sections (Additional file 1).

The first section gathered demographic information including sex, residency, age, occupational status, marital status, educational level, monthly income and number of children between six months and ten years. The second section gathered information about drugs, including questions about who is responsible for administering medication at home, whether their child had ever refused to take tablets/pills, what was done when their child did not like taking tablet drugs, whether the treatment process failed because their child did not like taking tablet drugs, whether their child ever refused to take liquid medications, what was done when their child did not like taking liquid drugs, whether the treatment process failed because their child did not like taking liquid medications and the source of information for drugs given to their child.

The third section gathered information about the child, including questions about whether the child had difficulties in swallowing medication, the type of swallowing problem, how many times they complained about swallowing difficulties, whether swallowing difficulties were discussed with a doctor, and the doctor’s recommendations.

The fourth section gathered information about practices, including questions about the tool had you used for giving a child their prescribed liquid medications, whether the leaflet attached to the drug was read, whether the child was given a dose higher than that prescribed to treat them more quickly, whether the child was given more than one type of oral medication at the same time, whether the child was given medications without prescription from a doctor, the type of medications used, whether the time the drugs were given to the child were recorded, and what was done with any remaining drugs when the child had recovered. They were also asked for the hours at which they would give their child medicine if it had been prescribed for three times a day.

A pilot study was undertaken with 30 parents to check for necessary modifications in the questionnaire, but as the selected parents all understood the questionnaire, no modifications were made. The questionnaire was reviewed and evaluated by experts in the field of pharmacy practice to ensure its content validity.

### Outcomes

The primary outcome of our study was a composite consisted of parents' practices during the administration of oral drugs to their children at home and the acceptance behaviors of their children. In addition, swallowing problems during the administration of these drugs were included in the composite outcome. As a secondary outcome, we reported the most commonly used self-therapies by parents for their children.

### Sample size

An online Raosoft sample size calculator (http://www.raosoft.com/samplesize.html( was applied to determine the sample size, which was 377. By assuming a response distribution for parents or caregivers who faced problems and difficulties when administering oral drugs to their children at home was 50%. A confidence level of 95% and a 5% margin of error were used, adding a non-response rate of 10% to increase accuracy.

### Statistics

The data was coded, categorised, and entered into the Statistical Package for the Social Sciences (SPSS), version 16.0. Descriptive statistics (e.g. frequency, percentage, mean, standard deviation) were used to illustrate the sociodemographic data and clinical data.

### Ethics

Ethical approval for the study was obtained from institutional review board (IRB) at An-Najah National University. The questionnaire content was described before the interview, and verbal informed consent was taken from each parent before the interviews were started.

## Results

### Sample characteristics

A total of 420 parents completed the survey giving a valid response rate of 96.8%. Table 1 provides the demographic information of the parents of our study. The mean age of parents was 30.2 with a standard deviation of 5.96; and the average number of children between 6 months and 10 years for each participant was 2.04 with a standard deviation of 0.98. Mothers constituted the majority of the parents (98.8%), most parents (86.4%) lived in the city, and (59.8%) had university education or higher. The majority of the parents had an income between 2000 and 4999 Shekels.

**Table 1:** Demographic information of parents (N = 420)

Characteristics	Item	Number (%)
**Gender**	Male	5 (1.2)
	Female	415 (98.8)
**Age**	<25	74 (17.6)
	25-29	135 (32)
	30-34	107 (25.5)
	35-39	73 (17.4)
	40-44	24 (5.7)
	>45	7 (1.7)
**Residency**	City	363 (86.4)
	Village	48 (11.4)
	Palestinian refugee camp	9 (2.1)
**Number of children aged between six months and ten years**	1 child	147 (35)
	2 children	147 (35)
	3 children	93 (22.1)
	4 children	27 (6.4)
	5 children	6 (1.4)
**Participant’s educational level**	Not educated	4 (1)
	Primary school	24 (5.7)
	Secondary school	141 (33.60
	University	251 (59.8)
**Father employment**^**a**^	Employed	406 (96.7)
	Non employed	10 (2.4)
**Mother employment**	Employed	83 (19.8)
	Non employed	337 (80.2)
**Income level of the family**^**b**^	<2000 **ILS**	102 (24.3)
	2000-4999 **ILS**	247 (58.8)
	5000-9999 **ILS**	56 (13.3)
	>10000 **ILS**	15 (3.6)
**Health insurance**	Governmental	219 (52.1)
	Private	58 (13.8)
	No insurance	143 (34)

^a^There are 4 dead fathers

^b^1**I**srae**l**i **S**hekel (**ILS**) equals 0.27 US Dollar

### Oral drug administration at home and acceptance behaviors of children

Ninety-three point three percent of those responsible for drug administration at home were mothers (Table 2). When asked about the acceptance behaviour of their children during oral medication administration, over half of those surveyed reported that they didn’t try to give their children tablets, sixty-four point two percent of those who had tried to do so reported that their children did not like taking tablet drugs (Table 2). When the children did not like taking tablet drugs, thirty-six point eight percent of the parents persuaded their children to drink more water, thirty-one point one percent requested another form of the drug and 30.2% crushed the capsule (Table 2).

**Table 2:** Oral drug administration at home and acceptance behaviors of children (*N* = 420)

Variable	Frequency (%)
**The person responsible for drug administration at home?**	
Father	6 (1.4)
Mother	392 (93.3)
Father & mother	21 (5)
Brother	1 (0.2)
**Did the child mind take oral pills?**	
Yes	106 (25.2)
No	59 (14)
Did not try it	255 (60.7)
**What did they do when the child refused to take tablet drugs?**^**a b**^	
Drink more water	39 (36.8)
Crush capsule	32 (30.2)
Open capsule	9 (8.5)
Break capsule	11 (10.4)
Change head position	6 (5.7)
Mix with food	8 (7.5)
Mix with milk	3 (2.8)
Dissolute in water or other drinks	32 (30.2)
Request another form	33 (31.1)
Stop drug	15 (14.2)
Give during sleep	3 (2.8)
**What did they do when the child refused to take liquid drugs?**^**a c**^	
Force child	153 (65.7)
Drink more water	57 (24.5)
Mix with milk	11 (4.7)
Mix with juice	50 (21.5)
Mix with food	12 (5.2)
Stop drug	26 (11.2)
Give during sleep	13 (5.6)
**Source of information about drugs**^**a**^	
Medical leaflet	216 (51.4)
Doctor	280 (66.7)
Nurse	5 (1.2)
Pharmacist	108 (25.7)
Ordinary people	11 (2.6)
Old experience	49 (11.7)
Internet	50 (11.9)

^**a**^Total exceeds 100 % as data are overlapping due to multiple answers

^b^Percentage was calculated by dividing by 106” the number of children refused taking capsules”

^c^Percentage was calculated by dividing by 233 “the number of the children refused taking liquid drugs “

All parents reported that they tried liquid medications, fifty-five point five percent reported that their children refused to take liquid medications, and when the child did not like taking liquid medications, almost two-thirds of the parents (65.7%) insisted their children take it anyway, twenty-four point five percent persuaded them to drink it with more water and 21.5% mixed it with juice (Table 2). There was a delay in treatment in 48.1% when the children did not like taking capsules or tablets, whereas it was 40.8% among those did not like taking liquid drugs.

As shown in Table 2, most parents (66.7%) obtained information about the medication from their doctors, 51.4% from medical leaflets and 25.7% from pharmacists.

### Swallowing problems during the administration of oral medications

Around 33.1% of those who were interviewed reported that their children had swallowing problems during the administration of oral medication, where vomiting was the most common one in 59% of the cases. Of those who reported swallowing problems, fifty-four percent discussed the problem with their doctor, who advised them to change the drug in most cases (48%) or offered advice to overcome the problem (38.7%); (Table 3).

**Table 3:** Swallowing problems influencing oral drug administration for managing childhood as reported by parents (*N0*= 139)^**a**^

Variable	Frequency (%)
**Type of problem**^**b**^	
Drugs hang in the throat	32 (23)
Uncomfortable sense	32 (23)
Chocking sense	18 (12.9)
Cough	32 (23)
Vomiting	82 (59)
**How many times did he complain of swallowing difficulty?**	
Always	29 (20.9)
Sometimes	109 (78.4)
One time	1 (0.7)
**Doctor advise about the problem**^**c**^	
Change drug	36 (48)
Change dose	1 (1.3)
Give some tips to overcome the problem	29 (38.7)
Forget the problem	9 (12)

^**a**^The number (139) reflected the number of children who had swallowing problems

^b^Total exceeds 100 % as data are overlapping due to multiple answers

^**c**^Percentage calculated by dividing by 75 “the number of parents discussed swallowing problem with their doctor”

### Parents' practices during the administration of oral drugs

Eighty-three point six percent of the parents used a syringe to administer oral liquid drugs; however, other tools were also used (Table 4). A minority of parents (12.4%) reported that they gave drugs in doses higher than prescribed by the doctor to treat their children more quickly. Forty-five percent of parents reported that they gave two drugs by mouth at the same time. Almost two-thirds of the parents (69%) said that they disposed of the residual amount of the drug when the child was recovered, while 30% kept it for later use.

**Table 4:** Parents’ practices during the administration of oral drugs (*N* = 420)

Variable	Frequency (%)
**A tool to give liquid drugs**^**a**^	
Cup attached with drug	39 (9.3)
Teaspoon	91 (21.7)
Tablespoon	30 (7.1)
Syringe	351 (83.6)
Other tools	4 (1)
**Reading leaflet**	
Yes	382 (91)
No	38 (9)
**Recording time when giving the drug**	
Yes	228 (54.3)
No	192 (45.7)

^**a**^Total exceeds 100 % as data are overlapping due to multiple answers

Surprisingly, ninety-one point nine percent of the parents used drugs without prescription from a doctor. The most commonly used self-therapies were antipyretics (n: 386, 100%), influenza drugs (n=142, 36.8%), cough drugs (*n*=109, 28.2%) and antibiotics (n: 102, 26.4%); (Table 5). In the final part of the survey, the parents were asked about the interval that should be left between each dose when a drug prescribed to be given three times daily, and it was revealed that 80.5% administered medication incorrectly.

**Table 5:** Types of self-therapies used by parents for their children^**a**^ (*N* = 386)

Variable	Frequency (%)
Antipyretics	386 (100)
Antibiotics	102 (26.4)
Antidiarrheal	37 (9.6)
Laxatives	10 (2.6)
Antiemetic	17 (4.4)
Cough drugs	109 (28.2)
Colic drugs	59 (15.3)
Creams	56 (15.5)
Influenza drugs	142 (36.8)

^**a**^Total exceeds 100 % as data are overlapping due to multiple answers

## Discussion

This study analyses the difficulties and errors made by parents when administering oral medication to their children at home. Erroneous practices have been discovered through our study, such as the use of inappropriate tools to give medicine to children, the use of over-the-counter medications, the administration of medications at incorrect intervals, and incorrect practices when the child did not like taking oral drugs, such as mixing the drugs with food or opening tablets.

A child’s adaptation to their drugs may be adversely affected by many factors, including the unpleasant taste [29], and this maladaptation could create difficulties for the parents when giving medications to their children. Around one-quarter of the parents in our study reported that their children did not like to take oral pills. Parents try to overcome the problem using many alternatives, such as mixing the drug with milk or with their children’s favourite food or juice. In our study, 7.5% of the parents tried mixing tablets with food, and 5.2% tried mixing the liquid drug with food. Drug efficacy and food absorption may be reduced when mixing drugs with certain foods [30, 31]. Twenty-one point five percent of the parents in our study tried mixing liquid medicines with juice, which may have adverse effects on the absorption, bioavailability and serum concentrations of some medicines. Kane and Lipsky [32] conducted a study about grapefruit-drug interaction, and reported that the serum concentrations of some drugs, such as cyclosporine, tacrolimus, and carbamazepine, were elevated if they interacted with grapefruit juice; these drugs have severe side effects depending on the dose, so the alteration of serum concentrations due to interaction with grapefruit juice may have side effects.

In recent years, Israel has grown and produced several forms of citrus fruit, e.g. a pummelo-grapefruit hybrid, named Israeli Jaffa Sweetie [33]. As we know, Israel ensures its products have free access to the Palestinian market [34]. In addition, Palestine is the chief competitor of the United States for exporting grapefruit during the winter months [35]. The importance of focusing on this point is that Palestine is one of the major grapefruit producing countries in the Middle East, in addition to the great availability of grapefruit juices in the Palestinian market. A collection of 60 drugs or more was established to have side effects if taken at the same time as, or even many hours after, taking a small amount of grapefruit juice [36].

Thirty point two percent of the participating parents with children who did not like taking tablets crushed the capsules in order to administer them to their children. Treatment effectiveness can be altered when crushing tablets, which may alter the absorption of the drug, therefore increasing or decreasing its serum level, which may lead to serious side effects [37]. Compounding in pharmacies is a skill that is often expected of children to solve problems and difficulties that parents or caregivers faced when administering oral drugs to their children at home. This condition poses a range of additional obstacles, including stabilization, palatability, compensation, and compounding legislation [13]. In addition, manufacturers need to be supported to develop new pediatric drug delivery systems such as mini-tablets [38]. Such advances have the ability to turn children's drug administration into healthier, more effective and appropriate [39].

Dosing errors in children are common, because dosing for children needs to be assessed individually based on many factors, such as the patient’s age and weight [40]. In this study, it was established that 21.7% of the participating parents used a teaspoon to give liquid medicine and 7.1% used a tablespoon, which may result in incorrect doses. Falagas et al [41] recommended that tablespoons and teaspoons should not be used due to their inaccuracy in dosing. In Palestine, sometimes, liquid drugs are not dispensed with a syringe or a quantitated cup so parents use spoons instead. In addition, 12.4% of the participating parents gave medicines with doses greater than that prescribed by the doctor in order to treat their children more quickly, which may in most cases lead to minor side effects, but also to hospitalisation or fatal side effects. Strenuous efforts are essential to prevent drug overdoses, which have recently become a leading cause of hospitalisation [42].

Surprisingly, when the participating parents were asked how to give a drug three times a day, only 19.5% know that it should be given every 8 hours, which means that the other 80.5% give the medications at incorrect intervals. Drug administration at incorrect intervals is a form of medication administration error. To ensure that serum drug levels are therapeutic, parents should administer drugs at the correct time [43].

This study has several limitations. The ability to generalise the study’s results to all Palestinians is limited because it was conducted only in Nablus. Different areas of Palestine should therefore be included in future studies for more representative results. Secondly, certain phenomena, such as the effect of the researcher being present when answering questions, may result in biases which cannot be controlled. Thirdly, this is a cross sectional study and causal relationships between variables could not be established. Fourthly, the use of convenience sampling may have led to bias in the conclusions. Lastly, the lack of information about age of the child is a major limitation for the current study which is important to distinguish between drug-sophisticated children (children who take medication frequently or chronically) versus drug-naive children (children who take medication infrequently) as to swallowing difficulties as there is good data demonstrating differences in age of tolerability of solid dosage forms between these two populations.

## Conclusions

This study has shown that there are a proportion of caregivers or parents who administer oral drugs to their children incorrectly, whether giving them at wrong intervals or doses, using incorrect instruments, or by administering non-prescription drugs. When children refuse to take their tablet or liquid medications, parents try to overcome the problem in many ways, such as mixing the drug with milk or with their children’s favourite food or juice, or by crushing tablets. This study also established that parents give medicines in doses that are higher than those prescribed by the doctor in order to treat their children more quickly. Increased awareness about medication errors is needed by parents. The development of educational programs that will provide parents with education about the practice of medication administration is thus recommended. Also, it is recommended that when a doctor prescribe a drug to be given many times daily, he should write the intervals between doses in hours like (x drug should be given every 8 hours not to write give it 3 times a day). The source of information about drugs should be the doctor and the pharmacist not the nurse, ordinary people, old experience or the internet. The last recommendation is that a law should be devised to force pharmacies not to dispense antibiotics unless prescribed by a doctor.

## Supplementary information

**Additional file 1:** Study questionnaires. This is the final English version of the questionnaire that was used to obtain data that helps to investigate the problems and difficulties that parents or caregivers faced when administering oral drugs to their children at home.

## Data Availability

The datasets used for the current study are available from the corresponding author upon request.

## Abbreviations

IRB: Institutional Review Board
OTC: Over-the-counter
SPSS: Statistical Package for the Social Sciences
