# Supplementary material for: The difficulties experienced during the preparation and administration of oral drugs by parents at home: a cross-sectional study from Palestine
Source: BMC Pediatr. 2020 May 7;20:198. doi: 10.1186/s12887-020-02105-w (PMC7204026; doi:10.1186/s12887-020-02105-w)
Supplement: Supplementary file 1 — Additional file 1: Study questionnaires. This is the final English version of the questionnaire that was used to obtain data that helps to investigate the problems and difficulties that parents or caregivers faced when administering oral drugs to their children at home. [file 12887_2020_2105_MOESM1_ESM.doc]

**Additional file 1: Study questionnaires.** This is the final English version of the questionnaire that was used to obtain data that helps to to investigate the problems and difficulties that parents or caregivers faced when administering oral drugs to their children at home.

Your participation in this survey is voluntary. You may choose not to participate. If you decide to participate in this survey, you may withdraw at any time. If you decide not to participate in this study, or if you withdraw from participating at any time you will not be penalized.

Filling out this form means that you accept to participate in this research.

**Part one: Demographic information**

1. Age: -----------
2. Gender: mother ( ) father( )
3. Residence: city ( ) village( ) camp( )
4. Number of children (6 months --- 10 years): ………………
5. Educational level:

Not educated ( ) primary school ( ) secondary school ( ) university ( )

1. Employment: father employment………… mother employment ………
2. Income level of the family in SHEKEL:

Less than 2000 2000-5000

5000-10000 more than 10000

1. Insurance: Governmental ( ) Private ( ) No insurance
2. How many times do you go to the doctor for your child annually? ……………….

**Part two: Information about drugs**

1. Who is responsible primarily for giving your child's medications at home?

- Father ( )
- Mother ( )
- Brother ( )
- Sister ( )
- Others ( )

# Did the child mind taking oral pills? Yes ( ) No ( ) Did not try it ( )

1. If (Yes), what did you do when he refused to take oral pills?

- Drink more water ( )
- Crush capsule ( )
- Open capsule ( )
- Break capsule ( )
- Change head position ( )
- Mix with food ( )
- Mix with milk ( )
- Dissolute in water or other drinks ( )
- Request another form ( )
- Stop drug ( )
- Give during sleep ( )

1. Did the treatment process fail because he refused to take the medication?

Yes ( ) No ( )

1. Did the child mind taking liquid drugs? Yes ( ) No ( ) Did not try it ( )
2. If (Yes), what did you do when he refused to take liquid drugs?

- Force the child to take it ( )
- Drink more water ( )
- Mix with milk ( )
- Mix with juice ( )
- Mix with food ( )
- Stop drug ( )
- Give during sleep ( )

1. Did the treatment process fail because he refused to take the medication?

Yes ( ) No ( )

1. Where / from whom do you get information about the medications that your child takes?

- Medical leaflet ( )
- Doctor ( )
- Nurse ( )
- Pharmacist ( )
- Ordinary people ( )
- Old experience ( )
- Internet ( )
- Others ( )

**Part three: Information about the child**

1. Did your child have a swallowing difficulty when he takes the drug?

Yes ( ) No ( )

**If Q18 was answered (Yes), answer questions (19-22)**

1. What is the type of that swallowing difficulty?

- Drugs hang in the throat ( )
- Uncomfortable sense ( )
- Chocking sense ( )
- Cough ( )
- Vomiting ( )

1. How many times did he complain of that swallowing difficulty?

Always ( ) Sometimes ( ) One time ( )

1. Did you discuss that difficulty with your doctor? Yes ( ) No ( )
2. If Q21 was answered (Yes), what did the doctor advice you?

- Change the drug ( )
- Change the dose ( )
- Give some tips to overcome the problem ( )
- Forget the problem ( )

**Part four: Information about the practice**

1. Which tool did you use for giving your child the prescribed liquid medications?

- Cup attached with drug ( )
- Teaspoon ( )
- Tablespoon ( )
- Syringe ( )
- Other tools ( )

1. Do you read the leaflet attached with the drug before giving medication to your children? Yes ( ) No ( )
2. Have you given your child a drug in doses more than prescribed by the doctor to treat him more quickly? Yes ( ) No ( )
3. Have you ever given your child more than one type of oral dugs in the same time?

Yes ( ) No ( )

1. Do you use drugs without prescription from a doctor?

Yes ( ) No ( )

1. If **Q27** was answered (Yes), what type of that drug?

- Antipyretics
- Antibiotics
- Antidiarrheal
- Laxatives
- Antiemetic
- Cough drugs
- Colic drugs
- Creams
- Influenza drugs

1. Did you record the time when giving the drugs to your children?

Yes ( ) No ( )

1. When the treatment process for your child completed, what do you do with the residual amount of liquid drugs?

- Keep it for later use
- Dispose it
- Give it to a relative

1. How do you give the medication 3 times a day? Can you give me an example of your hours?

---------------------------------------------------------------------------------------------------------------------------------------------------------------------------------------------------------------------------------------------------------------------------------------------------------------------------------------------------------------------------------------------------------------------------------------------------------------------------------------------------------------------------------------------------------------------------------------------------------

***Thank you***
